# The Use of Research Methods in Psychological Research: A Systematised Review

**DOI:** 10.3389/frma.2020.00001

**Published:** 2020-03-20

**Authors:** Salomé Elizabeth Scholtz, Werner de Klerk, Leon T. de Beer

**Affiliations:** ^1^Community Psychosocial Research (COMPRES), School of Psychosocial Health, North-West University, Potchefstroom, South Africa; ^2^WorkWell Research Institute, North-West University, Potchefstroom, South Africa

**Keywords:** research methods, research approach, research trends, psychological research, systematised review, research designs, research topic

## Abstract

Research methods play an imperative role in research quality as well as educating young researchers, however, the application thereof is unclear which can be detrimental to the field of psychology. Therefore, this systematised review aimed to determine what research methods are being used, how these methods are being used and for what topics in the field. Our review of 999 articles from five journals over a period of 5 years indicated that psychology research is conducted in 10 topics via predominantly quantitative research methods. Of these 10 topics, social psychology was the most popular. The remainder of the conducted methodology is described. It was also found that articles lacked rigour and transparency in the used methodology which has implications for replicability. In conclusion this article, provides an overview of all reported methodologies used in a sample of psychology journals. It highlights the popularity and application of methods and designs throughout the article sample as well as an unexpected lack of rigour with regard to most aspects of methodology. Possible sample bias should be considered when interpreting the results of this study. It is recommended that future research should utilise the results of this study to determine the possible impact on the field of psychology as a science and to further investigation into the use of research methods. Results should prompt the following future research into: a lack or rigour and its implication on replication, the use of certain methods above others, publication bias and choice of sampling method.

## Introduction

Psychology is an ever-growing and popular field (Gough and Lyons, 2016; Clay, 2017). Due to this growth and the need for science-based research to base health decisions on (Perestelo-Pérez, 2013), the use of research methods in the broad field of psychology is an essential point of investigation (Stangor, 2011; Aanstoos, 2014). Research methods are therefore viewed as important tools used by researchers to collect data (Nieuwenhuis, 2016) and include the following: quantitative, qualitative, mixed method and multi method (Maree, 2016). Additionally, researchers also employ various types of literature reviews to address research questions (Grant and Booth, 2009). According to literature, what research method is used and why a certain research method is used is complex as it depends on various factors that may include paradigm (O'Neil and Koekemoer, 2016), research question (Grix, 2002), or the skill and exposure of the researcher (Nind et al., 2015). How these research methods are employed is also difficult to discern as research methods are often depicted as having fixed boundaries that are continuously crossed in research (Johnson et al., 2001; Sandelowski, 2011). Examples of this crossing include adding quantitative aspects to qualitative studies (Sandelowski et al., 2009), or stating that a study used a mixed-method design without the study having any characteristics of this design (Truscott et al., 2010).

The inappropriate use of research methods affects how students and researchers improve and utilise their research skills (Scott Jones and Goldring, 2015), how theories are developed (Ngulube, 2013), and the credibility of research results (Levitt et al., 2017). This, in turn, can be detrimental to the field (Nind et al., 2015), journal publication (Ketchen et al., 2008; Ezeh et al., 2010), and attempts to address public social issues through psychological research (Dweck, 2017). This is especially important given the now well-known replication crisis the field is facing (Earp and Trafimow, 2015; Hengartner, 2018).

Due to this lack of clarity on method use and the potential impact of inept use of research methods, the aim of this study was to explore the use of research methods in the field of psychology through a review of journal publications. Chaichanasakul et al. (2011) identify reviewing articles as the opportunity to examine the development, growth and progress of a research area and overall quality of a journal. Studies such as Lee et al. (1999) as well as Bluhm et al. (2011) review of qualitative methods has attempted to synthesis the use of research methods and indicated the growth of qualitative research in American and European journals. Research has also focused on the use of research methods in specific sub-disciplines of psychology, for example, in the field of Industrial and Organisational psychology Coetzee and Van Zyl (2014) found that South African publications tend to consist of cross-sectional quantitative research methods with underrepresented longitudinal studies. Qualitative studies were found to make up 21% of the articles published from 1995 to 2015 in a similar study by O'Neil and Koekemoer (2016). Other methods in health psychology, such as Mixed methods research have also been reportedly growing in popularity (O'Cathain, 2009).

A broad overview of the use of research methods in the field of psychology as a whole is however, not available in the literature. Therefore, our research focused on answering what research methods are being used, how these methods are being used and for what topics in practice (i.e., journal publications) in order to provide a general perspective of method used in psychology publication. We synthesised the collected data into the following format: *research topic* [areas of scientific discourse in a field or the current needs of a population (Bittermann and Fischer, 2018)], *method* [data-gathering tools (Nieuwenhuis, 2016)], *sampling* [elements chosen from a population to partake in research (Ritchie et al., 2009)], *data collection* [techniques and research strategy (Maree, 2016)], and *data analysis* [discovering information by examining bodies of data (Ktepi, 2016)]. A systematised review of recent articles (2013 to 2017) collected from five different journals in the field of psychological research was conducted.

## Methods

### Design

Grant and Booth (2009) describe systematised reviews as the review of choice for post-graduate studies, which is employed using some elements of a systematic review and seldom more than one or two databases to catalogue studies after a comprehensive literature search. The aspects used in this systematised review that are similar to that of a systematic review were a full search within the chosen database and data produced in tabular form (Grant and Booth, 2009).

### Sampling

Sample sizes and timelines vary in systematised reviews (see Lowe and Moore, 2014; Pericall and Taylor, 2014; Barr-Walker, 2017). With no clear parameters identified in the literature (see Grant and Booth, 2009), the sample size of this study was determined by the purpose of the sample (Strydom, 2011), and time and cost constraints (Maree and Pietersen, 2016). Thus, a non-probability purposive sample (Ritchie et al., 2009) of the top five psychology journals from 2013 to 2017 was included in this research study. Per Lee (2015) American Psychological Association (APA) recommends the use of the most up-to-date sources for data collection with consideration of the context of the research study. As this research study focused on the most recent trends in research methods used in the broad field of psychology, the identified time frame was deemed appropriate.

Psychology journals were only included if they formed part of the top five English journals in the miscellaneous psychology domain of the Scimago Journal and Country Rank (Scimago Journal & Country Rank, 2017). The Scimago Journal and Country Rank provides a yearly updated list of publicly accessible journal and country-specific indicators derived from the Scopus® database (Scopus, 2017b) by means of the Scimago Journal Rank (SJR) indicator developed by Scimago from the algorithm Google PageRank™ (Scimago Journal & Country Rank, 2017). Scopus is the largest global database of abstracts and citations from peer-reviewed journals (Scopus, 2017a). Reasons for the development of the Scimago Journal and Country Rank list was to allow researchers to assess scientific domains, compare country rankings, and compare and analyse journals (Scimago Journal & Country Rank, 2017), which supported the aim of this research study. Additionally, the goals of the journals had to focus on topics in psychology in general with no preference to specific research methods and have full-text access to articles.

The following list of top five journals in 2018 fell within the abovementioned inclusion criteria (1) Australian Journal of Psychology, (2) British Journal of Psychology, (3) Europe's Journal of Psychology, (4) International Journal of Psychology and lastly the (5) Journal of Psychology Applied and Interdisciplinary.

Journals were excluded from this systematised review if no full-text versions of their articles were available, if journals explicitly stated a publication preference for certain research methods, or if the journal only published articles in a specific discipline of psychological research (for example, industrial psychology, clinical psychology etc.).

### Procedure

**Figure 1:**
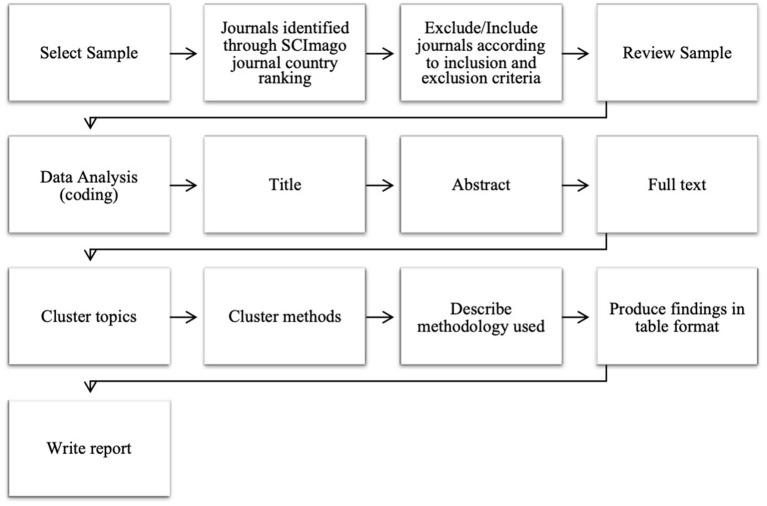
Systematised review procedure.

The researchers followed a procedure (see Figure 1) adapted from that of Ferreira et al. (2016) for systematised reviews. Data collection and categorisation commenced on 4 December 2017 and continued until 30 June 2019. All the data was systematically collected and coded manually (Grant and Booth, 2009) with an independent person acting as co-coder. Codes of interest included the research topic, method used, the design used, sampling method, and methodology (the method used for data collection and data analysis). These codes were derived from the wording in each article. Themes were created based on the derived codes and checked by the co-coder. Lastly, these themes were catalogued into a table as per the systematised review design.

### Rigour

According to Johnston et al. (2019), “literature screening, selection, and data extraction/analyses” (p. 7) are specifically tailored to the aim of a review. Therefore, the steps followed in a systematic review must be reported in a comprehensive and transparent manner. The chosen systematised design adhered to the rigour expected from systematic reviews with regard to full search and data produced in tabular form (Grant and Booth, 2009). The rigorous application of the systematic review is, therefore discussed in relation to these two elements.

Firstly, to ensure a comprehensive search, this research study promoted review transparency by following a clear protocol outlined according to each review stage before collecting data (Johnston et al., 2019). This protocol was similar to that of Ferreira et al. (2016) and approved by three research committees/stakeholders and the researchers (Johnston et al., 2019). The eligibility criteria for article inclusion was based on the research question and clearly stated, and the process of inclusion was recorded on an electronic spreadsheet to create an evidence trail (Bandara et al., 2015; Johnston et al., 2019). Microsoft Excel spreadsheets are a popular tool for review studies and can increase the rigour of the review process (Bandara et al., 2015). Screening for appropriate articles for inclusion forms an integral part of a systematic review process (Johnston et al., 2019). This step was applied to two aspects of this research study: the choice of eligible journals and articles to be included. Suitable journals were selected by the first author and reviewed by the second and third authors. Initially, all articles from the chosen journals were included. Then, by process of elimination, those irrelevant to the research aim, i.e., interview articles or discussions etc., were excluded.

To ensure rigourous data extraction, data was first extracted by one reviewer, and an independent person verified the results for completeness and accuracy (Johnston et al., 2019). The research question served as a guide for efficient, organised data extraction (Johnston et al., 2019). Data was categorised according to the codes of interest, along with article identifiers for audit trails such as authors, title and aims of articles. The categorised data was based on the aim of the review (Johnston et al., 2019) and synthesised in tabular form under methods used, how these methods were used, and for what topics in the field of psychology.

## Results

**Figure 2:**
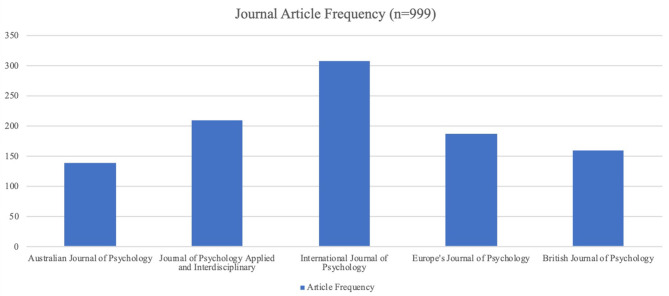
Journal article frequency.

The initial search produced a total of 1,145 articles from the 5 journals identified. Inclusion and exclusion criteria resulted in a final sample of 999 articles (Figure 2). Articles were co-coded into 84 codes, from which 10 themes were derived (Table 1).

**Table 1:** Codes used to form themes (research topics).

**Theme**	**Code amount**	**Codes**
Social Psychology	31	Aggression SP, Attitude SP, Belief SP, Child abuse SP, Conflict SP, Culture SP, Discrimination SP, Economic, Family illness, Family, Group, Help, Immigration, Intergeneration, Judgement, Law, Leadership, Marriage SP, Media, Optimism, Organisational and Social justice, Parenting SP, Politics, Prejudice, Relationships, Religion, Romantic Relationships SP, Sex and attraction, Stereotype, Violence, Work
Experimental Psychology	17	Anxiety, stress and PTSD, Coping, Depression, Emotion, Empathy, Facial research, Fear and threat, Happiness, Humor, Mindfulness, Mortality, Motivation and Achievement, Perception, Rumination, Self, Self-efficacy
Cognitive Psychology	12	Attention, Cognition, Decision making, Impulse, Intelligence, Language, Math, Memory, Mental, Number, Problem solving, Reading
Health Psychology	7	Addiction, Body, Burnout, Health, Illness (Health Psychology), Sleep (Health Psychology), Suicide and Self-harm
Physiological Psychology	6	Gender, Health (Physiological psychology), Illness (Physiological psychology), Mood disorders, Sleep (Physiological psychology), Visual research
Developmental Psychology	3	Attachment, Development, Old age
Personality	3	Machiavellian, Narcissism, Personality
Psychological Psychology	3	Programme, Psychology practice, Theory
Education and Learning	1	Education and Learning
Psychometrics	1	Measure
Code Total	84	

**Figure 3:**
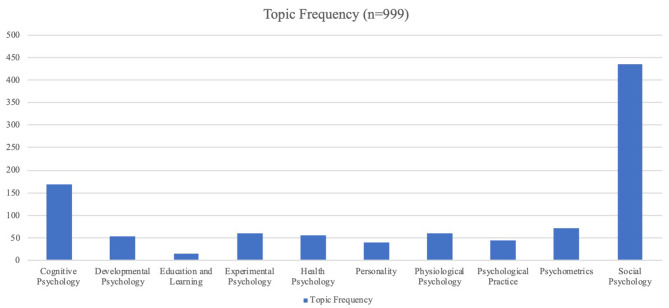
Topic frequency (international sample).

These 10 themes represent the *topic* section of our research question (Figure 3). All these topics except, for the final one, *psychological practice*, were found to concur with the research areas in psychology as identified by Weiten (2010). These research areas were chosen to represent the derived codes as they provided broad definitions that allowed for clear, concise categorisation of the vast amount of data. Article codes were categorised under particular themes/topics if they adhered to the research area definitions created by Weiten (2010). It is important to note that these areas of research do not refer to specific disciplines in psychology, such as industrial psychology; but to broader fields that may encompass sub-interests of these disciplines.

In the case of *developmental psychology*, researchers conduct research into human development from childhood to old age. *Social psychology* includes research on behaviour governed by social drivers. Researchers in the field of *educational psychology* study how people learn and the best way to teach them. *Health psychology* aims to determine the effect of psychological factors on physiological health. *Physiological psychology*, on the other hand, looks at the influence of physiological aspects on behaviour. *Experimental psychology* is not the only theme that uses experimental research and focuses on the traditional core topics of psychology (for example, sensation). *Cognitive psychology* studies the higher mental processes. *Psychometrics* is concerned with measuring capacity or behaviour. *Personality* research aims to assess and describe consistency in human behaviour (Weiten, 2010). The final theme of *psychological practice* refers to the experiences, techniques, and interventions employed by practitioners, researchers, and academia in the field of psychology.

Articles under these themes were further subdivided into methodologies: method, sampling, design, data collection, and data analysis. The categorisation was based on information stated in the articles and not inferred by the researchers. Data were compiled into two sets of results presented in this article. The first set addresses the aim of this study from the perspective of the topics identified. The second set of results represents a broad overview of the results from the perspective of the methodology employed. The second set of results are discussed in this article, while the first set is presented in table format. The discussion thus provides a broad overview of methods use in psychology (across all themes), while the table format provides readers with in-depth insight into methods used in the individual themes identified. We believe that presenting the data from both perspectives allow readers a broad understanding of the results. Due a large amount of information that made up our results, we followed Cichocka and Jost (2014) in simplifying our results. Please note that the numbers indicated in the table in terms of methodology differ from the total number of articles. Some articles employed more than one method/sampling technique/design/data collection method/data analysis in their studies.

What follows is the results for *what methods are used, how these methods are used, and which topics in psychology they are applied to*. Percentages are reported to the second decimal in order to highlight small differences in the occurrence of methodology.

**Figure 4:**
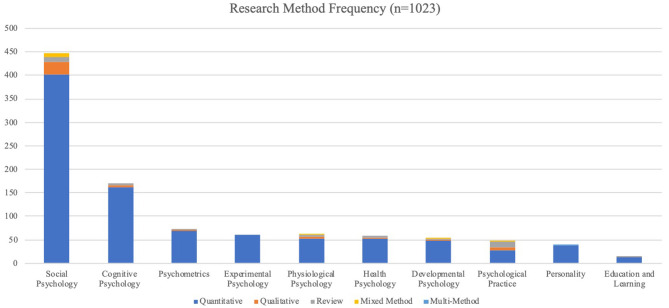
Research method frequency in topics.

Firstly, with regard to the *research methods* used, our results show that researchers are more likely to use quantitative research methods (90.22%) compared to all other research methods. Qualitative research was the second most common research method but only made up about 4.79% of the general method usage. Reviews occurred almost as much as qualitative studies (3.91%), as the third most popular method. Mixed-methods research studies (0.98%) occurred across most themes, whereas multi-method research was indicated in only one study and amounted to 0.10% of the methods identified. The specific use of each method in the topics identified is shown in Table 2 and Figure 4.

**Table 2:** Research methods in psychology.

**Research Method**	**Social Psychology**	**Cognitive Psychology**	**Psychometrics**	**Experimental Psychology**	**Physiological Psychology**	**Health Psychology**	**Developmental Psychology**	**Psychological Practice**	**Personality**	**Education and Learning**
Quantitative	401	162	69	60	52	52	48	28	38	13
Qualitative	28	4	1	0	5	2	3	5	0	1
Review	11	5	2	0	3	4	1	13	0	1
Mixed Methods	7	0	0	0	1	0	1	1	0	0
Multi-method	0	0	0	0	0	0	0	0	1	0
Total	447	171	72	60	61	58	53	47	39	15

Secondly, in the case of *how these research methods are employed*, our study indicated the following.

**Figure 5:**
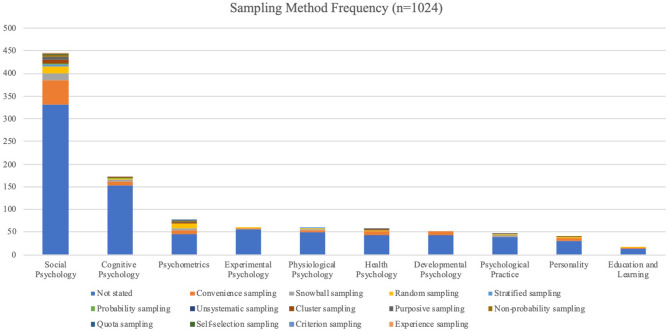
Sampling method frequency in topics.

*Sampling*−78.34% of the studies in the collected articles did not specify a sampling method. From the remainder of the studies, 13 types of sampling methods were identified. These sampling methods included broad categorisation of a sample as, for example, a probability or non-probability sample. General samples of convenience were the methods most likely to be applied (10.34%), followed by random sampling (3.51%), snowball sampling (2.73%), and purposive (1.37%) and cluster sampling (1.27%). The remainder of the sampling methods occurred to a more limited extent (0–1.0%). See Table 3 and Figure 5 for sampling methods employed in each topic.

**Table 3:** Sampling use in the field of psychology.

**Sampling Method**	**Social Psychology**	**Cognitive Psychology**	**Psychometrics**	**Experimental Psychology**	**Physiological Psychology**	**Health Psychology**	**Developmental Psychology**	**Psychological Practice**	**Personality**	**Education and Learning**
Not stated	331	153	45	57	49	43	43	38	31	14
Convenience sampling	55	8	10	1	6	8	9	2	6	1
Random sampling	15	3	9	1	2	2	0	2	1	1
Snowball sampling	14	4	4	1	2	0	0	3	0	0
Purposive sampling	6	0	2	0	0	2	0	3	1	0
Cluster sampling	8	1	2	0	0	2	0	0	0	0
Stratified sampling	4	1	2	0	1	1	0	0	0	0
Non-probability sampling	4	0	1	0	0	0	0	0	1	0
Probability sampling	3	1	0	0	0	0	0	0	0	0
Quota sampling	1	0	1	0	0	0	0	0	0	0
Criterion sampling	1	0	0	0	0	0	0	0	0	0
Self-selection sampling	1	0	0	0	0	0	0	0	0	0
Unsystematic sampling	0	1	0	0	0	0	0	0	0	0
Total	443	172	76	60	60	58	52	48	40	16

*Designs* were categorised based on the articles' statement thereof. Therefore, it is important to note that, in the case of quantitative studies, non-experimental designs (25.55%) were often indicated due to a lack of experiments and any other indication of design, which, according to Laher (2016), is a reasonable categorisation. Non-experimental designs should thus be compared with experimental designs only in the description of data, as it could include the use of correlational/cross-sectional designs, which were not overtly stated by the authors. For the remainder of the research methods, “not stated” (7.12%) was assigned to articles without design types indicated.

**Figure 6:**
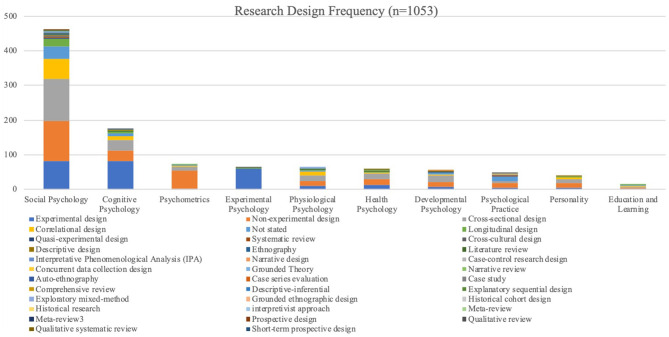
Design frequency in topics.

From the 36 identified designs the most popular designs were cross-sectional (23.17%) and experimental (25.64%), which concurred with the high number of quantitative studies. Longitudinal studies (3.80%), the third most popular design, was used in both quantitative and qualitative studies. Qualitative designs consisted of ethnography (0.38%), interpretative phenomenological designs/phenomenology (0.28%), as well as narrative designs (0.28%). Studies that employed the review method were mostly categorised as “not stated,” with the most often stated review designs being systematic reviews (0.57%). The few mixed method studies employed exploratory, explanatory (0.09%), and concurrent designs (0.19%), with some studies referring to separate designs for the qualitative and quantitative methods. The one study that identified itself as a multi-method study used a longitudinal design. Please see how these designs were employed in each specific topic in Table 4, Figure 6.

**Table 4:** Design use in the field of psychology.

**Research Design**	**Social Psychology**	**Cognitive Psychology**	**Psychometrics**	**Experimental Psychology**	**Physiological Psychology**	**Health Psychology**	**Developmental Psychology**	**Psychological Practice**	**Personality**	**Education and Learning**
Experimental design	82	82	3	60	10	12	8	6	4	3
Non-experimental design	115	30	51	0	13	17	13	13	14	3
Cross-sectional design	123	31	12	1	19	17	21	5	13	2
Correlational design	56	12	3	0	10	2	2	0	4	2
Not stated	37	7	3	0	4	2	4	14	1	3
Longitudinal design	21	6	2	1	1	2	2	0	2	3
Quasi-experimental design	4	1	0	0	0	0	2	1	0	0
Systematic review	3	0	0	0	1	1	0	1	0	0
Cross-cultural design	3	0	0	1	0	0	0	1	0	0
Descriptive design	2	0	0	0	0	0	3	0	0	0
Ethnography	4	0	0	0	0	0	0	0	0	0
Literature review	1	1	0	0	1	1	0	0	0	0
Interpretative Phenomenological Analysis (IPA)	2	0	0	0	1	0	0	0	0	0
Narrative design	1	0	0	0	0	0	1	1	0	0
Case-control research design	0	0	0	0	0	2	0	0	0	0
Concurrent data collection design	1	0	0	0	1	0	0	0	0	0
Grounded Theory	1	0	0	0	1	0	0	0	0	0
Narrative review	0	1	0	0	0	1	0	0	0	0
Auto-ethnography	1	0	0	0	0	0	0	0	0	0
Case series evaluation	0	0	0	0	0	0	0	1	0	0
Case study	1	0	0	0	0	0	0	0	0	0
Comprehensive review	0	1	0	0	0	0	0	0	0	0
Descriptive-inferential	0	0	0	0	0	0	0	0	1	0
Explanatory sequential design	1	0	0	0	0	0	0	0	0	0
Exploratory mixed-method	0	0	0	0	1	0	0	1	0	0
Grounded ethnographic design	0	1	0	0	0	0	0	0	0	0
Historical cohort design	0	1	0	0	0	0	0	0	0	0
Historical research	0	0	0	0	0	0	0	1	0	0
interpretivist approach	0	0	0	0	0	0	0	1	0	0
Meta-review	1	0	0	0	0	0	0	1	0	0
Prospective design	1	0	0	0	0	0	0	0	0	0
Qualitative review	0	0	0	0	0	0	0	1	0	0
Qualitative systematic review	0	0	0	0	0	1	0	0	0	0
Short-term prospective design	0	1	0	0	0	0	0	0	0	0
Total	461	175	74	63	63	58	56	48	39	16

**Figure 7:**
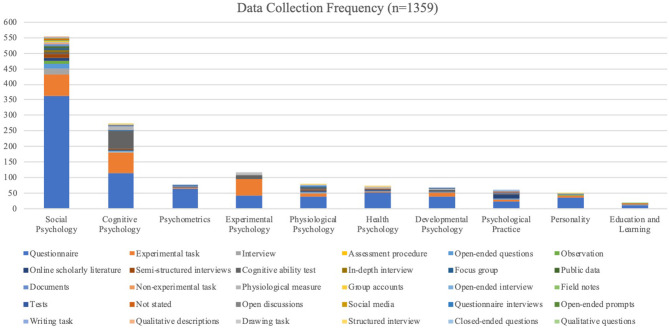
Data collection frequency in topics.

*Data collection and analysis*—data collection included 30 methods, with the data collection method most often employed being questionnaires (57.84%). The experimental task (16.56%) was the second most preferred collection method, which included established or unique tasks designed by the researchers. Cognitive ability tests (6.84%) were also regularly used along with various forms of interviewing (7.66%). Table 5 and Figure 7 represent data collection use in the various topics. Data analysis consisted of 3,857 occurrences of data analysis categorised into ±188 various data analysis techniques shown in Table 6 and Figures 1–7. Descriptive statistics were the most commonly used (23.49%) along with correlational analysis (17.19%). When using a qualitative method, researchers generally employed thematic analysis (0.52%) or different forms of analysis that led to coding and the creation of themes. Review studies presented few data analysis methods, with most studies categorising their results. Mixed method and multi-method studies followed the analysis methods identified for the qualitative and quantitative studies included.

**Table 5:** Data collection in the field of psychology.

**Data Collection**	**Social Psychology**	**Cognitive Psychology**	**Psychometrics**	**Experimental Psychology**	**Physiological Psychology**	**Health Psychology**	**Developmental Psychology**	**Psychological Practice**	**Personality**	**Education and Learning**
Questionnaire	364	113	65	42	40	51	39	24	37	11
Experimental task	68	66	3	52	9	5	11	5	5	1
Cognitive ability test	9	57	1	12	6	1	5	1	1	0
Physiological measure	3	12	1	6	2	5	3	0	1	0
Interview	19	3	0	1	3	0	2	2	0	1
Online scholarly literature	10	4	0	0	3	4	0	10	0	0
Open-ended questions	15	3	0	1	3	1	2	3	0	0
Semi-structured interviews	10	3	0	0	3	2	1	2	0	1
Observation	10	1	0	0	0	0	0	0	2	0
Documents	5	1	1	0	0	0	0	1	2	0
Focus group	6	1	2	0	1	0	0	0	0	0
Not stated	2	1	1	0	0	0	1	4	0	1
Public data	6	1	0	0	0	0	0	2	0	1
Drawing task	0	2	0	1	1	1	0	2	0	0
In-depth interview	6	0	0	0	1	0	0	0	0	0
Structured interview	0	2	0	0	1	2	0	0	1	0
Writing task	1	0	0	0	4	0	0	1	0	0
Questionnaire interviews	1	0	1	0	2	0	1	0	0	0
Non-experimental task	4	0	0	0	0	0	0	0	0	0
Tests	2	2	0	0	0	0	0	0	0	0
Group accounts	2	0	0	0	0	0	0	1	0	0
Open-ended prompts	1	1	0	0	0	0	0	1	0	0
Field notes	2	0	0	0	0	0	0	0	0	0
Open-ended interview	2	0	0	0	0	0	0	0	0	0
Qualitative questions	0	0	0	0	0	1	0	0	0	1
Social media	1	0	0	0	0	0	0	0	1	0
Assessment procedure	0	0	0	1	0	0	0	0	0	0
Closed-ended questions	0	0	0	0	0	0	0	1	0	0
Open discussions	1	0	0	0	0	0	0	0	0	0
Qualitative descriptions	1	0	0	0	0	0	0	0	0	0
Total	551	273	75	116	79	73	65	60	50	17

**Table 6:** Data analysis in the field of psychology.

**Data Analysis**	**Social Psychology**	**Cognitive Psychology**	**Psychometrics**	**Experimental Psychology**	**Physiological Psychology**	**Health Psychology**	**Developmental Psychology**	**Psychological Practice**	**Personality**	**Education and Learning**
Not stated	5	1	2	0	0	1	1	5	0	1
Actor-Partner Interdependence Model (APIM)	4	0	0	0	0	0	0	0	0	0
Analysis of Covariance (ANCOVA)	17	8	1	3	4	2	1	0	0	1
Analysis of Variance (ANOVA)	112	60	16	29	15	17	15	6	5	3
Auto-regressive path coefficients	0	0	1	0	0	0	0	0	0	0
Average variance extracted (AVE)	1	0	0	0	0	1	0	0	0	0
Bartholomew's classification system	1	0	0	0	0	0	0	0	0	0
Bayesian analysis	3	0	0	0	1	0	0	0	0	0
Bibliometric analysis	1	1	0	0	0	0	0	1	0	0
Binary logistic regression	1	1	0	0	1	4	1	0	0	0
Binary multilevel regression	0	0	0	1	0	0	0	0	0	0
Binomial and Bernoulli regression models	2	0	0	0	0	0	0	0	0	0
Binomial mixed effects model	1	0	0	0	0	0	0	0	0	0
Bivariate Correlations	32	10	3	0	4	3	5	1	1	1
Bivariate logistic correlations	1	0	0	0	0	1	0	0	0	0
Bootstrapping	39	16	2	3	5	1	6	1	2	1
Canonical correlations	0	0	0	0	0	0	0	0	2	0
Cartesian diagram	1	0	0	0	0	0	0	0	0	0
Case-wise diagnostics	0	1	0	0	0	0	1	0	0	0
Casual network analysis	0	0	0	1	0	0	0	0	0	0
Categorisation	5	2	0	0	1	1	0	4	0	0
Categorisation of responses	2	0	0	0	0	0	0	0	0	0
Category codes	3	1	0	0	0	1	0	0	0	0
Cattell's scree-test	0	0	1	0	0	0	0	0	0	0
Chi-square tests	52	20	17	5	6	11	8	7	4	3
Classic Parallel Analysis (PA)	0	0	1	0	0	1	0	0	1	0
Cluster analysis	7	0	0	0	1	1	1	1	0	1
Coded	15	3	1	2	1	1	1	2	1	0
Cohen d effect size	14	5	2	1	3	2	3	1	0	1
Common method variance (CMV)	5	0	1	0	0	0	0	0	0	0
Comprehensive Meta-Analysis (CMA)	0	0	0	0	0	0	0	0	1	0
Confidence Interval (CI)	2	0	0	0	0	1	0	0	0	0
Confirmatory Factor Analysis (CFA)	57	13	40	0	2	4	7	1	3	1
Content analysis	9	1	0	0	2	1	0	1	0	0
Convergent validity	1	0	0	0	0	0	0	0	0	0
Cook's distance	0	1	0	0	1	0	0	0	0	0
Correlated-trait-correlated-method minus one model	1	0	0	0	0	0	0	0	0	0
Correlational analysis	259	85	44	18	27	31	34	8	33	8
Covariance matrix	3	0	1	0	0	0	0	0	0	0
Covariance modelling	0	1	1	0	0	0	0	0	0	0
Covariance structure analyses	2	0	0	0	0	0	0	0	0	0
Cronbach's alpha	61	14	18	6	5	10	8	3	7	5
Cross-validation	0	0	2	0	0	0	0	0	0	1
Cross-lagged analyses	1	2	1	0	0	0	1	0	0	0
Dependent t-test	1	2	0	0	1	1	0	1	0	0
Descriptive statistics	324	132	43	49	41	43	36	28	29	10
Differentiated analysis	0	0	0	0	0	0	1	0	0	0
Discriminate analysis	1	0	2	0	0	0	0	0	0	1
Discursive psychology	1	0	0	0	0	0	0	0	0	0
Dominance analysis	1	0	0	0	0	0	0	0	0	0
Expectation maximisation	2	1	0	0	0	0	0	1	0	0
Exploratory data Analysis	1	1	0	0	1	1	0	0	0	0
Exploratory Factor Analysis (EFA)	14	5	24	0	1	1	4	0	4	0
Exploratory structural equation modelling (ESEM)	0	0	1	0	0	0	0	0	1	0
Factor analysis	12	4	16	0	2	1	5	0	2	0
Measurement invariance testing	0	0	0	0	0	0	0	0	0	0
Four-way mixed ANOVA	0	1	0	1	0	0	0	0	0	0
Frequency rate	20	1	4	2	1	2	2	2	0	0
Friedman test	1	0	0	0	0	0	0	0	0	0
Games-Howell *post hoc*	2	2	0	0	0	1	0	0	0	0
General linear model analysis	1	2	0	0	0	0	1	1	0	0
Greenhouse-Geisser correction	2	5	0	0	0	0	1	1	1	1
Grounded theory method	0	0	0	0	0	0	0	0	0	1
Grounded theory methodology using open and axial coding	1	0	0	0	0	0	0	0	0	0
Guttman split-half	0	0	1	0	0	0	0	0	0	0
Harman's one-factor test	13	2	0	0	0	1	2	0	0	0
Herman's criteria of experience categorisation	0	0	0	0	0	0	0	1	0	0
Hierarchical CFA (HCFA)	0	0	1	0	0	0	0	0	0	0
Hierarchical cluster analysis	1	0	0	0	0	0	0	0	0	0
Hierarchical Linear Modelling (HLM)	76	22	2	3	7	6	7	4	4	1
Huynh-Felt correction	1	0	0	0	0	0	0	0	0	0
Identified themes	3	0	0	0	1	0	0	0	0	0
Independent samples t-test	38	9	4	4	4	8	3	3	1	1
Inductive open coding	1	0	0	0	0	0	0	0	0	0
Inferential statistics	2	0	0	0	0	0	1	0	0	0
Interclass correlation	3	0	1	0	0	0	0	0	0	0
Internal consistency	3	1	2	0	0	0	0	0	0	0
Interpreted and defined	0	0	0	0	1	0	0	0	0	0
Interpretive Phenomenological Analysis (IPA)	2	1	0	0	1	0	0	0	0	0
Item fit analysis	1	0	5	0	0	0	0	0	0	0
K-means clustering	0	0	0	0	0	0	0	1	0	0
Kaiser-meyer-Olkin measure of sampling adequacy	2	0	8	0	0	0	2	0	2	0
Kendall's coefficients	3	1	0	0	0	0	0	0	0	0
Kolmogorov-Smirnov test	1	2	1	1	2	2	0	0	1	0
Lagged-effects multilevel modelling	1	1	0	0	0	0	0	0	0	0
Latent class differentiation (LCD)	1	0	0	0	0	0	0	0	0	0
Latent cluster analysis	0	0	0	0	0	1	0	0	0	0
Latent growth curve modelling (LGCM)	1	0	0	0	0	0	0	1	1	0
Latent means	1	0	0	0	0	0	0	0	0	0
Latent Profile Analysis (LPA)	1	1	0	0	0	0	0	0	0	0
Linear regressions	69	19	4	10	3	12	5	3	13	0
Linguistic Inquiry and Word Count	0	0	0	0	1	0	0	0	0	0
Listwise deletion method	0	0	0	0	0	1	0	0	0	0
Log-likelihood ratios	0	0	0	0	0	1	0	0	0	0
Logistic mixed-effects model	1	0	0	0	0	0	0	0	0	0
Logistic regression analyses	17	0	1	0	4	2	1	0	0	1
Loglinear Model	2	0	0	0	0	0	0	0	0	0
Mahalanobis distances	0	2	0	0	0	1	0	0	0	0
Mann-Whitney U tests	6	4	2	1	2	0	2	4	0	0
Mauchly's test	0	1	0	2	0	0	0	1	0	1
Maximum likelihood method	11	3	9	0	1	3	2	3	1	0
Maximum-likelihood factor analysis with promax rotation	0	1	0	0	0	0	0	0	0	0
Measurement invariance testing	4	1	1	0	1	0	0	0	0	0
Mediation analysis	29	7	1	2	4	3	5	0	3	0
Meta-analysis	3	0	1	0	0	0	0	1	0	0
Microanalysis	1	0	0	0	0	0	0	0	0	0
Minimum significant difference (MSD) *post hoc* comparison	0	1	0	0	0	0	0	0	0	0
Mixed ANOVAs	19	6	0	10	1	2	1	4	1	0
Mixed linear model	0	0	0	1	0	0	1	0	0	0
Mixed-design ANCOVA	1	1	0	0	0	0	0	0	0	0
Mixed-effects multiple regression models	1	0	0	0	0	0	0	0	0	0
Moderated hierarchical regression model	1	0	0	0	0	0	0	0	0	0
Moderated regression analysis	8	4	0	0	1	0	1	0	1	0
Monte Carlo Markov Chains	2	0	1	0	0	0	0	0	0	0
Multi-group analysis	3	0	0	0	0	0	0	0	0	0
Multidimensional Random Coefficient Multinomial Logit (MRCML)	0	0	1	0	0	0	0	0	0	0
Multidimensional Scaling	2	0	0	0	0	0	0	0	0	0
Multiple-Group Confirmatory Factor Analysis (MGCFA)	3	0	0	0	0	2	0	0	0	0
Multilevel latent class analysis	1	0	0	0	0	1	0	0	0	0
Multilevel modelling	7	2	1	1	1	0	0	1	1	0
Multilevel Structural Equation Modelling (MSEM)	2	0	0	0	0	0	0	0	0	0
Multinominal logistic regression (MLR)	1	0	0	0	0	0	0	0	0	0
Multinominal regression analysis	1	0	0	0	0	2	0	0	0	0
Multiple Indicators Multiple Causes (MIMIC)	0	0	0	0	1	1	0	0	0	0
Multiple mediation analysis	2	6	0	0	2	2	1	0	0	0
Multiple regression	34	15	3	0	3	4	5	0	7	2
Multivariate analysis of co-variance (MANCOVA)	12	2	1	1	0	1	1	0	1	0
Multivariate Analysis of Variance (MANOVA)	38	8	4	5	5	6	9	1	1	2
Multivariate hierarchical linear regression	1	1	0	0	0	0	0	0	0	0
Multivariate linear regression	0	1	0	0	0	0	1	0	0	0
Multivariate logistic regression analyses	1	0	0	0	0	0	0	0	0	0
Multivariate regressions	2	1	0	0	0	0	1	0	0	0
Nagelkerke's R square	0	0	0	0	0	1	0	0	0	0
Narrative analysis	1	0	0	0	0	0	1	0	0	0
Negative binominal regression with log link	0	0	0	0	0	1	0	0	0	0
Newman-Keuls	0	1	0	0	0	1	0	0	0	0
Nomological Validity Analysis	0	0	1	0	0	0	0	0	0	0
One sample t-test	8	10	1	7	4	6	4	0	1	0
Ordinary Least-Square regression (OLS)	2	2	0	1	0	0	0	0	0	0
Pairwise deletion method	0	0	0	0	0	1	0	0	0	0
Pairwise parameter comparison	4	0	0	0	0	0	2	0	0	0
Parametric Analysis	0	0	0	1	0	0	0	0	0	0
Partial Least Squares regression method (PLS)	1	1	0	0	0	0	0	0	0	0
Path analysis	21	9	0	1	2	4	5	1	2	0
Path-analytic model test	1	0	0	0	0	0	0	0	0	0
Phenomenological analysis	0	0	1	0	0	0	0	1	0	0
Polynomial regression analyses	1	0	0	0	0	0	0	0	0	0
*post-hoc* Fisher LSD	0	1	0	0	0	0	0	0	0	0
Principal axis factoring	2	1	4	0	0	0	1	0	0	0
Principal component analysis (PCA)	8	1	12	1	1	0	3	2	5	1
Pseudo-panel regression	1	0	0	0	0	0	0	0	0	0
Quantitative content analysis	0	0	0	0	1	0	0	0	0	0
Receiver operating characteristic (ROC) curve analysis	2	0	0	1	0	0	0	0	0	0
Relative weight analysis	1	0	0	0	0	0	0	0	0	0
Repeated measures analyses of variances (rANOVA)	18	22	1	7	5	2	1	1	1	1
Ryan-Einot-Gabriel-Welsch multiple F test	1	0	0	0	0	0	0	0	0	0
Satorra-Bentler scaled chi-square statistic	0	0	3	0	0	0	0	0	0	0
Scheffe's test	3	0	0	0	0	1	0	0	0	0
Sequential multiple mediation analysis	1	0	0	0	0	0	0	0	0	0
Shapiro-Wilk test	2	3	0	2	1	0	0	0	0	0
Sobel Test	13	5	0	1	0	2	4	0	0	0
Squared multiple correlations	1	0	0	0	0	0	0	0	0	0
Squared semi-partial correlations (sr2)	2	0	0	0	0	0	0	0	0	0
Stepwise regression analysis	3	2	0	0	1	0	0	0	2	0
Structural Equation Modelling (SEM)	56	22	3	3	3	5	5	0	5	3
Structure analysis	0	0	0	0	0	0	1	0	0	0
Subsequent t-test	0	0	0	0	1	0	0	0	0	0
Systematic coding- Gemeinschaft-oriented	1	0	0	0	1	0	0	0	0	0
Task analysis	2	0	0	0	0	0	0	0	0	0
Thematic analysis	11	2	0	0	3	0	2	2	0	0
Three (condition)-way ANOVA	0	4	0	0	1	0	1	0	0	0
Three-way hierarchical loglinear analysis	0	2	0	0	0	0	0	0	0	0
Tukey-Kramer corrections	0	0	0	1	0	1	0	0	0	0
Two-paired sample t-test	7	6	1	1	0	3	1	1	0	1
Two-tailed related t-test	0	1	1	0	1	0	0	0	0	0
Unadjusted Logistic regression analysis	0	1	0	0	0	0	0	0	0	0
Univariate generalized linear models (GLM)	2	0	0	0	0	0	0	0	0	0
Variance inflation factor (VIF)	3	1	0	0	0	0	0	0	1	0
Variance-covariance matrix	1	0	0	0	0	0	0	1	0	0
Wald test	1	1	0	0	0	0	0	0	0	0
Ward's hierarchical cluster method	0	0	0	0	0	0	0	0	0	1
Weighted least squares with corrections to means and variances (WLSMV)	2	0	0	0	0	0	0	0	0	0
Welch and Brown-Forsythe F-ratios	0	1	0	0	0	1	0	0	0	0
Wilcoxon signed-rank test	3	3	0	2	0	0	0	2	0	1
Wilks' Lamba	6	0	0	0	0	0	1	0	0	0
Word analysis	0	0	0	0	0	0	0	1	0	0
Word Association Analysis	1	0	0	0	0	0	0	0	0	0
*z* scores	5	6	1	0	1	1	0	1	0	0
Total	1738	635	329	192	198	237	225	117	152	55

Results of the topics researched in psychology can be seen in the tables, as previously stated in this article. It is noteworthy that, of the 10 topics, social psychology accounted for 43.54% of the studies, with cognitive psychology the second most popular research topic at 16.92%. The remainder of the topics only occurred in 4.0–7.0% of the articles considered. A list of the included 999 articles is available under the section “View Articles” on the following website: https://methodgarden.xtrapolate.io/. This website was created by Scholtz et al. (2019) to visually present a research framework based on this Article's results.

## Discussion

This systematised review categorised full-length articles from five international journals across the span of 5 years to provide insight into the use of research methods in the field of psychology. Results indicated what methods are used how these methods are being used and for what topics (why) in the included sample of articles. The results should be seen as providing insight into method use and by no means a comprehensive representation of the aforementioned aim due to the limited sample. To our knowledge, this is the first research study to address this topic in this manner. Our discussion attempts to promote a productive way forward in terms of the key results for method use in psychology, especially in the field of academia (Holloway, 2008).

With regard to the methods used, our data stayed true to literature, finding only common research methods (Grant and Booth, 2009; Maree, 2016) that varied in the degree to which they were employed. Quantitative research was found to be the most popular method, as indicated by literature (Breen and Darlaston-Jones, 2010; Counsell and Harlow, 2017) and previous studies in specific areas of psychology (see Coetzee and Van Zyl, 2014). Its long history as the first research method (Leech et al., 2007) in the field of psychology as well as researchers' current application of mathematical approaches in their studies (Toomela, 2010) might contribute to its popularity today. Whatever the case may be, our results show that, despite the growth in qualitative research (Demuth, 2015; Smith and McGannon, 2018), quantitative research remains the first choice for article publication in these journals. Despite the included journals indicating openness to articles that apply any research methods. This finding may be due to qualitative research still being seen as a new method (Burman and Whelan, 2011) or reviewers' standards being higher for qualitative studies (Bluhm et al., 2011). Future research is encouraged into the possible biasness in publication of research methods, additionally further investigation with a different sample into the proclaimed growth of qualitative research may also provide different results.

Review studies were found to surpass that of multi-method and mixed method studies. To this effect Grant and Booth (2009), state that the increased awareness, journal contribution calls as well as its efficiency in procuring research funds all promote the popularity of reviews. The low frequency of mixed method studies contradicts the view in literature that it's the third most utilised research method (Tashakkori and Teddlie's, 2003). Its' low occurrence in this sample could be due to opposing views on mixing methods (Gunasekare, 2015) or that authors prefer publishing in mixed method journals, when using this method, or its relative novelty (Ivankova et al., 2016). Despite its low occurrence, the application of the mixed methods design in articles was methodologically clear in all cases which were not the case for the remainder of research methods.

Additionally, a substantial number of studies used a combination of methodologies that are not mixed or multi-method studies. Perceived fixed boundaries are according to literature often set aside, as confirmed by this result, in order to investigate the aim of a study, which could create a new and helpful way of understanding the world (Gunasekare, 2015). According to Toomela (2010), this is not unheard of and could be considered a form of “structural systemic science,” as in the case of qualitative methodology (observation) applied in quantitative studies (experimental design) for example. Based on this result, further research into this phenomenon as well as its implications for research methods such as multi and mixed methods is recommended.

Discerning how these research methods were applied, presented some difficulty. In the case of sampling, most studies—regardless of method—did mention some form of inclusion and exclusion criteria, but no definite sampling method. This result, along with the fact that samples often consisted of students from the researchers' own academic institutions, can contribute to literature and debates among academics (Peterson and Merunka, 2014; Laher, 2016). Samples of convenience and students as participants especially raise questions about the generalisability and applicability of results (Peterson and Merunka, 2014). This is because attention to sampling is important as inappropriate sampling can debilitate the legitimacy of interpretations (Onwuegbuzie and Collins, 2017). Future investigation into the possible implications of this reported popular use of convenience samples for the field of psychology as well as the reason for this use could provide interesting insight, and is encouraged by this study.

Additionally, and this is indicated in Table 6, articles seldom report the research designs used, which highlights the pressing aspect of the lack of rigour in the included sample. Rigour with regards to the applied empirical method is imperative in promoting psychology as a science (American Psychological Association, 2020). Omitting parts of the research process in publication when it could have been used to inform others' research skills should be questioned, and the influence on the process of replicating results should be considered. Publications are often rejected due to a lack of rigour in the applied method and designs (Fonseca, 2013; Laher, 2016), calling for increased clarity and knowledge of method application. Replication is a critical part of any field of scientific research and requires the “complete articulation” of the study methods used (Drotar, 2010, p. 804). The lack of thorough description could be explained by the requirements of certain journals to only report on certain aspects of a research process, especially with regard to the applied design (Laher, 20). However, naming aspects such as sampling and designs, is a requirement according to the APA's Journal Article Reporting Standards (JARS-Quant) (Appelbaum et al., 2018). With very little information on how a study was conducted, authors lose a valuable opportunity to enhance research validity, enrich the knowledge of others, and contribute to the growth of psychology and methodology as a whole. In the case of this research study, it also restricted our results to only reported samples and designs, which indicated a preference for certain designs, such as cross-sectional designs for quantitative studies.

Data collection and analysis were for the most part clearly stated. A key result was the versatile use of questionnaires. Researchers would apply a questionnaire in various ways, for example in questionnaire interviews, online surveys, and written questionnaires across most research methods. This may highlight a trend for future research.

With regard to the topics these methods were employed for, our research study found a new field named “psychological practice.” This result may show the growing consciousness of researchers as part of the research process (Denzin and Lincoln, 2003), psychological practice, and knowledge generation. The most popular of these topics was social psychology, which is generously covered in journals and by learning societies, as testaments of the institutional support and richness social psychology has in the field of psychology (Chryssochoou, 2015). The APA's perspective on 2018 trends in psychology also identifies an increased amount of psychology focus on how social determinants are influencing people's health (Deangelis, 2017).

This study was not without limitations and the following should be taken into account. Firstly, this study used a sample of five specific journals to address the aim of the research study, despite general journal aims (as stated on journal websites), this inclusion signified a bias towards the research methods published in these specific journals only and limited generalisability. A broader sample of journals over a different period of time, or a single journal over a longer period of time might provide different results. A second limitation is the use of Excel spreadsheets and an electronic system to log articles, which was a manual process and therefore left room for error (Bandara et al., 2015). To address this potential issue, co-coding was performed to reduce error. Lastly, this article categorised data based on the information presented in the article sample; there was no interpretation of what methodology could have been applied or whether the methods stated adhered to the criteria for the methods used. Thus, a large number of articles that did not clearly indicate a research method or design could influence the results of this review. However, this in itself was also a noteworthy result. Future research could review research methods of a broader sample of journals with an interpretive review tool that increases rigour. Additionally, the authors also encourage the future use of systematised review designs as a way to promote a concise procedure in applying this design.

## Conclusion

Our research study presented the use of research methods for published articles in the field of psychology as well as recommendations for future research based on these results. Insight into the complex questions identified in literature, regarding what methods are used how these methods are being used and for what topics (why) was gained. This sample preferred quantitative methods, used convenience sampling and presented a lack of rigorous accounts for the remaining methodologies. All methodologies that were clearly indicated in the sample were tabulated to allow researchers insight into the general use of methods and not only the most frequently used methods. The lack of rigorous account of research methods in articles was represented in-depth for each step in the research process and can be of vital importance to address the current replication crisis within the field of psychology. Recommendations for future research aimed to motivate research into the practical implications of the results for psychology, for example, publication bias and the use of convenience samples.

## Ethics Statement

This study was cleared by the North-West University Health Research Ethics Committee: NWU-00115-17-S1.

## Author Contributions

All authors listed have made a substantial, direct and intellectual contribution to the work, and approved it for publication.

### Conflict of Interest

The authors declare that the research was conducted in the absence of any commercial or financial relationships that could be construed as a potential conflict of interest.
